# Chronic sensory contact with subordinated conspecifics promotes splenic glucocorticoid resistance in experimentally wounded C57BL/6N male mice

**DOI:** 10.1038/s41598-024-61581-7

**Published:** 2024-05-13

**Authors:** Jessica Schiele, Giulia Mazzari, Antonia Struck, Yorick Bailer, Dominik Langgartner, Stefan O. Reber

**Affiliations:** Laboratory for Molecular Psychosomatics, Department of Psychosomatic Medicine and Psychotherapy, Ulm University Medical Center, Albert-Einstein-Allee 11, 89081 Ulm, Baden-Württemberg Germany

**Keywords:** Wounding, Chronic subordinate colony housing (CSC), Chronic psychosocial stress, Sensory contact, Glucocorticoid resistance, Inflammation, Stress and resilience

## Abstract

Chronic psychosocial stress induced by the chronic subordinate colony housing (CSC, 19 Days) paradigm promotes functional splenic in vitro glucocorticoid (GC) resistance, but only if associated with significant bite wounding or prior abdominal transmitter implantation. Moreover, sensory contact to social defeat of conspecifics represents a social stressor for the observer individual. As the occurence and severity of bite wounding is not adequately controllable, the present study aimed to develop an animal model, allowing a bite wound-independent, more reliable generation of chronically-stressed mice characterized by functional splenic in vitro GC resistance. Therefore, male C57BL/6N mice received a standardized sterile intraperitoneal (i.p.) incision surgery or SHAM treatment one week prior to 19-days of (i) CSC, (ii) witnessing social defeat during CSC exposure in sensory contact (SENS) or (iii) single-housing for control (SHC), before assessing basal and LPS-induced splenic in vitro cell viability and GC resistance*.* Our results indicate that individually-housed SENS but not CSC mice develop mild signs of splenic in vitro GC resistance, when undergoing prior i.p.-wounding. Taken together and considering that future studies are warranted, our findings support the hypothesis that the combination of repeated standardized i.p.-wounding with chronic sensory stress exposure represents an adequate tool to induce functional splenic in vitro GC resistance independent of the occurrence of uncontrollable bite wounds required in social stress paradigms to induce a comparable phenotype.

## Introduction

Chronic psychosocial stress is a risk factor for many psychosomatic disorders, characterized by an over(re)active immune system and chronic low-grade inflammation^1–6^. Many clinical and preclinical studies^7–13^ suggest that stress-associated inflammation is at least partly promoted via development of glucocorticoid (GC) resistance. The latter is defined as a state of diminished sensitivity of certain immune cell subpopulations to the anti-inflammatory action of GCs^8,10^. Specifically, myeloid CD11b^+^ cells seem to play a critical role in this context^11,12,14^. In a series of preclinical experiments employing the chronic subordinate colony housing (CSC) paradigm as an acknowledged model for social stress-associated posttraumatic stress disorder (PTSD) in male mice^15,16^ we could show that particularly CD11b^+^Ly6G^+^Ly6C^+^ polymorphonuclear (PMN)-myeloid-derived suppressor cells (MDSCs) play a critical role in psychosocial stress-induced GC resistance^17–20^. In detail, toll-like receptor (TLR)2, but not TLR4, upregulation and increased basal and lipopolysaccharide (LPS)-induced in vitro cell viability indicated priming^21^ and activation of stress-induced PMN-MDSCs, neutrophils, and monocytes/ monocyte-like (MO)-MDSCs locally in the bone marrow (BM)^17^. Subsequently, these cells emigrate into the periphery and, if psychosocial stress is accompanied by significant physical injury (bite wounding), accumulate in the spleen^17^, where PMN-MDSCs and monocytes/MO-MDSCs upregulate TLR4 expression. This promotes NF-κB hyperactivation upon LPS-stimulation exclusively in PMN-MDSCs, thereby exceeding the anti-inflammatory capacities of GCs and resulting in GC resistance^17^. However, given that the occurrence of bite wounds and, consequently, the development of CSC-induced splenic functional in vitro GC resistance is dependent on both the aggressiveness of the chosen dominant aggressor CD-1 mouse as well as the coping behavior shown by each C57BL/6N CSC mouse^18^ and, thus, can’t be properly controlled for by the experimenter, it is extremely difficult to reliably generate CSC mice with a certain severity of bite wounds and functional splenic in vitro GC resistance. Of particular importance in the context of the latter, we showed earlier that abdominal transmitter implantation (G2 HR E-Mitter; Starr Life Science Corp., Oakmont, PA; USA) one week prior to CSC exposure also in the absence of bite wounds enabled CSC to promote splenomegaly, basal and LPS-induced in vitro splenocyte activation and insensitivity towards GC in in vitro LPS-treated splenocytes^22^. The latter supports the hypothesis that not only severe bite wounds but also wounding induced by experimental surgery is able to promote resistance towards immunosuppressive GCs in chronically stressed mice.

Of further importance for the current study, not only individuals personally experiencing severely stressful situations can get traumatized, also individuals witnessing traumatization of others can develop symptoms of PTSD^23,24^. The latter is in line with studies reporting an endogenous salivary cortisol response in individuals observing others performing the Trier Social Stress Test, consisting of a public speaking and mental arithmetic task in front of an interview jury^25^. Furthermore, preclinical rodent models allowing sensory contact of experimental animals with conspecifics receiving direct physical defeat revealed that also witnessing traumatization of others results in typical signs of chronic stress^26^, including adrenal hypertrophy, thymus involution and elevated plasma corticosterone (CORT) concentrations^27,28^. In support of sustained sensory contact with an experienced stressor having profound negative effects on the observer’s stress recovery, we recently showed that CSC mice housed in sensory contact with the last dominant CD-1 aggressor used during CSC exposure recovered slower from CSC-induced diurnal dysrhythmia than CSC mice without subsequent sensory contact with the last aggressor^22^.

Therefore, it was the aim of the present study to develop a modified version of the CSC paradigm, allowing the bite wound-independent and, thus, more reliable generation of chronically-stressed mice characterized by functional splenic in vitro GC resistance. To achieve this aim, we housed male mice individually in visual, auditory and olfactory contact (SENS) with a CSC colony over 19 consecutive days and compared them with single-housed control (SHC) mice, with all experimental SHC, SENS and CSC mice undergoing either a standardized sterile i.p. incision surgery or a respective SHAM treatment (i.e., anesthesia without wounding) one week prior to the start of the 19-day experimental procedure. Of note, as we have recently shown that repeated i.g. administrations of *Mycobacterium vaccae* NCTC 11659 dissolved in borate-buffered saline (BBS) prior to CSC exposure were protective against CSC-induced functional splenic in vitro GC resistance in bitten CSC mice^29^, all mice in the present study have been repeatedly administered i.g. with BBS prior to CSC or SENS. The latter would have allowed us in a second set of mice to assess whether repeated i.g. administrations with *M. vaccae* NCTC 11659 are protective also against CSC and/or SENS-induced splenic GC resistance in unbitten but experimentally wounded mice. However, given the absent/mild effects of CSC/SENS on splenic GC resistance in i.p.-wounded mice, we abstained from completing the *M. vaccae* NCTC 11659 groups in this particular setup for animal welfare reasons.

## Results

### Effects of prior i.p. incision surgery on CSC- and SENS-induced physiological changes

**Figure 1:**
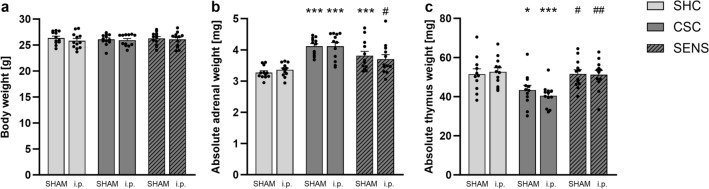
Effects on body, absolute adrenal and thymus weight. After termination of the experiment on Day 20, body (**a**), absolute adrenal (**b**) and thymus (**c**) weight of each experimental mouse was determined. Data are presented as bars (mean + SEM) including individual values. SHC, CSC and SENS groups for SHAM and i.p.: *N* = 12. *P ≤ 0.05, ***P ≤ 0.001 versus (vs.) respective SHC; ^#^*P* ≤ 0.05, ^##^*P* ≤ 0.01 vs. respective CSC. *i.p.* intraperitoneally wounded group, *SHC* single-housed control mice, *CSC* chronic subordinate colony housing, *SENS* experimental mice housed in sensory contact with a CSC colony, *SEM* standard error of mean.

Statistical analysis using Mann–Whitney *U* (MWU) comparisons did not reveal significant differences in absolute body weight on Day 20 of CSC between the groups (Fig. 1a).

Chronic subordinate colony housing resulted in significantly increased absolute adrenal weight (Fig. 1b) in both SHAM (MWU: *P* < 0.001) and i.p.-wounded mice (MWU: *P* < 0.001) when compared to respective SHC mice. Although SENS vs. SHC significantly or by trend increased absolute adrenal weight in SHAM (MWU: *P* = 0.001) or i.p.-wounded (MWU: *P* = 0.07) mice, respectively, adrenal glands of both SHAM-SENS (MWU: *P* = 0.06) and i.p.-SENS (MWU: *P* = 0.029) mice were by trend or significantly lighter than the ones of respective CSC mice (Fig. 1b). Regarding the absolute thymus weight, a main effect for factor stress (two-way ANOVA: F(stress)_2,65_ = 13.35; *P* = 0.001) was found, with CSC significantly reducing absolute thymus weight compared with SHC mice in both the SHAM (Bonferroni: *P* = 0.039) and i.p. (Bonferroni: *P* < 0.001) group, and SENS increasing absolute thymus weight compared to CSC mice in both the SHAM (Bonferroni: *P* = 0.029) and i.p. (Bonferroni: *P* = 0.003) group (Fig. 1c).

### Effects of prior i.p. incision surgery on CSC- and SENS-induced spleen changes

**Figure 2:**
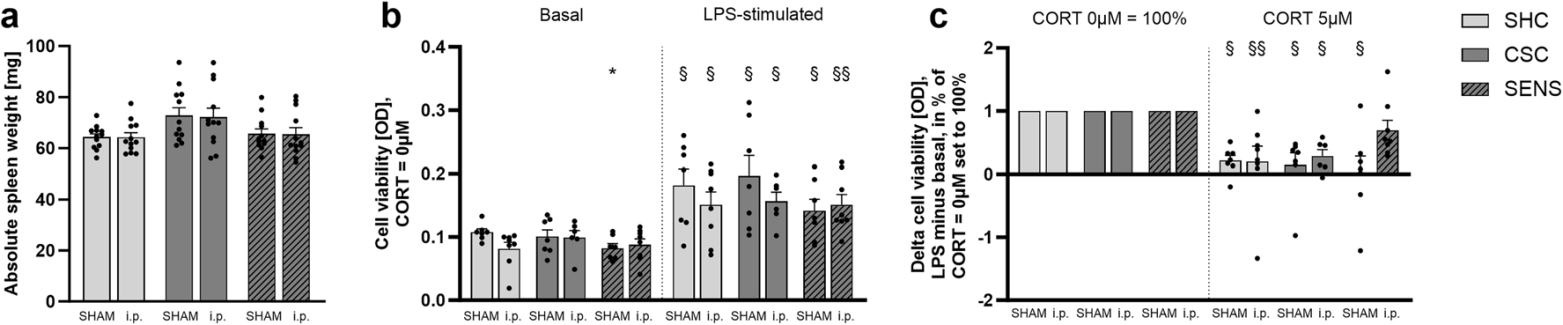
Effects on spleen weight and splenic in vitro GC sensitivity. After termination of the experiment on Day 20, absolute spleen weight (**a**) in vitro splenic cell viability under unstimulated (basal) or LPS-stimulated conditions (**b**), as well as relative splenic delta cell viability (LPS minus basal; 0 µM CORT set to 100%) in response to 5 µM CORT (**c**) was assessed. Data are presented as bars (mean + SEM) including individual values. (a) SHC, CSC and SENS groups for SHAM and i.p.: *N* = 12; (b+c) SHAM-SHC: *N* = 7, i.p.-SHC: *N* = 8; SHAM-CSC: *N* = 7, i.p.-CSC: *N* = 6, SHAM-SENS: *N* = 7, i.p.-SENS: *N* = 8. *P ≤ 0.05 versus (vs.) respective SHC; ^§^*P* ≤ 0.05, ^§§^*P* ≤ 0.01 vs. respective basal (CORT 0 µM = 100%). *i.p.* intraperitoneally wounded group, *SHC* single-housed control mice, *CSC* chronic subordinate colony housing, *SENS* experimental mice housed in sensory contact with a CSC colony, *OD* optical density, *LPS* lipopolysaccharide, *CORT* corticosterone, *SEM* standard error of mean.

Statistical analysis revealed a main effect of factor stress for absolute spleen weight (two-way ANOVA: F(stress)_2,66_ = 6.324; *P* = 0.003), with only SHAM-CSC mice by trend showing an increase in absolute spleen weight compared with respective SHC males (Bonferroni: *P* = 0.055; Fig. 2a). Bite wound severity was not found to be different between SHAM- (20.14 ± 3.789) and i.p.-wounded (23.17 ± 5.924) CSC mice (MWU).

Statistical analysis using MWU comparisons further revealed that the cell viability of isolated spleen cells from SHAM-SENS mice was significantly lower compared with respective SHC mice (*P* = 0.024) when cultured in vitro in the absence of LPS. Moreover, spleen cell viability of all groups was significantly increased when cultured in vitro in the presence vs. absence of LPS (Wilcoxon; SHAM-SHC: *P* = 0.047; i.p.-SHC: *P* = 0.016; SHAM-CSC: *P* = 0.016; i.p.-CSC: *P* = 0.031; SHAM-SENS: *P* = 0.031; i.p.-SENS: *P* = 0.008; Fig. 2b). Delta cell viability (LPS minus basal condition, 0 µM CORT set to 100%) of isolated splenocytes cultured in vitro in the presence (i.e., 5 µM) of CORT was significantly reduced in all experimental groups (Wilcoxon; SHAM-SHC: *P* = 0.016; i.p.-SHC: *P* = 0.008; SHAM-CSC: *P* = 0.016; i.p.-CSC: *P* = 0.031; SHAM-SENS: *P* = 0.031), except the i.p.-SENS group. In line with the latter, in the presence of 5 µM CORT, i.p.-SENS mice by trend showed an increased delta cell viability compared with respective i.p.-CSC mice (MWU: *P* = 0.059; Fig. 2c).

## Discussion

CSC-induced development of functional splenic in vitro GC resistance is critically dependent on the occurrence of significant bite wounding during CSC exposure^17,18,20,22^, with the latter representing an experimentally not adequately controllable factor. Therefore, it was the aim of the present study to develop a modified version of the CSC paradigm, allowing the bite wound-independent and, thus, more reliable generation of chronically-stressed mice characterized by functional splenic in vitro GC resistance. As CSC mice undergoing abdominal transmitter implantation one week prior to CSC reliably develop functional splenic in vitro GC resistance even in absence of bite wounds^19^, any kind of physical injury, either in form of received bite wounds during stressor exposure or in form of a planned and controllable surgery prior to stressor exposure seems to be able to promote chronic stress-induced GC resistance. Moreover, clinical^23–25^ and preclinical^22,26–28^ data support the hypothesis that witnessing conspecifics when suffering stress or trauma represents a reliable stressor for the observer individual. Therefore, we in the current study tested the hypothesis that individually-housed male mice living in visual, auditory and olfactory contact (SENS) with a CSC colony will develop functional splenic in vitro GC resistance, when undergoing a standardized sterile i.p. incision surgery but not a respective SHAM treatment (i.e., anesthesia without wounding) one week prior to the start of a 19-day SENS exposure.

In line with own previous studies^16,30,31^, CSC mice of both the SHAM and i.p.-wounded group developed adrenal enlargement and thymus involution, which is the most predictive biomarker for classification and class prediction in the CSC paradigm^26^ and a typical sign of chronic stress in rodents^16,32–35^. These findings convincingly indicate that the CSC paradigm worked reliably in the current study, independent of SHAM or i.p. wounding one week prior to the start of the stress procedure. Interestingly and in line with our hypothesis, absolute adrenal weight in the current study was increased also in SENS mice of the SHAM and i.p. (by trend) wounding group, when compared to respective SHC mice, confirming that sensory contact with an adjacent CSC colony represents a reliable stressor for the observer individuals. However, as SENS in contrast to CSC mice in both the SHAM and i.p. wounding group did not develop thymus involution when compared with respective SHC mice, this data suggests that witnessing the social defeat of conspecifics is not as stressful as being socially defeated itself. More precisely, we hypothesize that SENS mice, in contrast to CSC mice, are not characterized by chronic activation of the sympathetic nervous system. The latter is based on the facts that (i) the medullary part of the thymus expresses high densities of beta-adrenergic receptors^36^, which are involved in a cAMP-mediated thymocyte apoptosis and the consequent decrease of thymocyte numbers^37–39^, and that CSC mice (ii) develop thymus atrophy even when adrenalectomized prior to CSC, (iii) show increased plasma NE levels following 19 days of CSC exposure, (iv) show increased basal plasma morning CORT concentrations only transiently following the initiation of CSC on Day 1 and again on Day 8 (1st resident change) and (iv) develop basal evening hypocorticism, despite CSC-induced thymus involution is present continuously throughout CSC exposure^16,40–42^.

With respect to the spleen, earlier own findings^17,18,20,43^ and findings reported by others using the social disruption stress (SDR) paradigm^44^ indicate that severe bite wounds are critically required for a stress-induced splenomegaly, increase in basal and LPS-induced in vitro splenocyte viability and functional splenic in vitro GC resistance. In line with these findings, the mild or absent bite wounds in SHAM-wounded CSC and SENS mice, respectively, in the present study were not paralleled by significant enlargement of spleen weights, increased basal or LPS-induced in vitro splenocyte viability and functional splenic in vitro GC resistance. Noteworthy, as reported previously^17,18,20,43^ LPS was able to increase splenocyte viability in all experimental groups assessed. Moreover, as a bite score of about 20 has been identified in previous own studies as critical threshold in terms of CSC-induced splenic invasion of CD11b^+^ cells and, thus, development of splenomegaly and functional splenic in vitro GC resistance^17,18^, the statistical main effect of stress on spleen weight and the trend towards an increase in spleen weight in the SHAM-CSC vs. respective SHC group found in the present study is in line with bite scores of slightly above 20 in both CSC groups. Importantly and in contrast to our earlier findings indicating that an abdominal transmitter implantation one week prior to CSC was able to promote CSC-induced splenomegaly, an increased basal or LPS-induced in vitro splenocyte viability and functional splenic in vitro GC resistance in unbitten CSC mice^19^, a standardized i.p. incision surgery prior to CSC exposure did not promote any of these spleen effects. One possible explanation might be that the sterile i.p. incision surgery one week prior to the start of the CSC paradigm was less invasive and almost recovered at the time when the animals were introduced into the CSC paradigm, which is in sharp contrast to the E-mitter implantation surgery resulting in a foreign object being present in the abdominal cavity during the whole CSC procedure. Thus, it might be that CSC in i.p.-wounded mice of the present study transiently induced the above reported typical effects on spleen weight and splenocyte function, but that these were not any more detectable at the end of the 20-day CSC procedure. In support of CSC-induced functional splenic in vitro GC resistance to represent only a transient phenomenon, isolated spleen cells from CSC mice 30 days following termination of the CSC paradigm were found to be sensitive to the immunosuppressive effects of CORT^43^. Therefore, future studies are warranted assessing whether repeated i.p.-wounding surgeries, best applied at the days of CSC assigned to changing the dominant aggressor mice (i.e., Days 1, 8 and 15), are able to promote the typical spleen effects seen in bitten CSC mice on Day 20 of CSC. Given that the latter start to develop around one week after the start of the CSC paradigm^43^, another possibility would be to assess spleen weight, basal or LPS-induced in vitro splenocyte viability and functional splenic in vitro GC resistance in unbitten CSC mice undergoing only one standardized i.p. incision surgery prior to CSC exposure around Day 8/9 of CSC exposure, and not on Day 20 as has been done in the current study.

Strikingly and in line with our hypothesis, i.p.-wounded but not SHAM SENS mice developed mild sings of functional splenic in vitro GC resistance despite not showing splenomegaly or increased basal and LPS-induced in vitro splenocyte viability. The latter was indicated by a significantly lower delta (LPS-basal; 0 µM CORT set to 100%) in vitro cell viability in SHAM SENS but not i.p.-wounded SENS mice compared with respective SHC mice in the presence of 5 µM CORT. Moreover, delta (LPS-basal; 0 µM CORT set to 100%) in vitro cell viability in i.p.-wounded SENS mice was by trend increased compared with SHAM-SENS mice in the presence of 5 µM CORT. One explanation for the fact that functional splenic in vitro GC resistance was detectable in i.p.-wounded mice exposed to 19 days of SENS but not 19 days of CSC might be that experimental mice during CSC exposure are housed in groups of four (plus one dominant aggressor mouse), whereas experimental mice during SENS are housed individually, preventing any kind of social contact with other subordinate conspecifics while experiencing chronic stress. The latter hypothesis is supported by data showing that the time spent in close proximity with conspecifics, also known as huddling or defensive aggregation, is increased in rats exposed as quad to a threatening cat odor stimulus (i.e., predator odor)^45^. In turn, and this is of particular importance for the interpretation of the current findings, it has been shown that the inhibition of grooming and the increase in immobility in the presence of cat odor was significantly more pronounced for rats exposed to the stressor alone than in a quad^46^. In line with the latter, rats exposed to cat odor alone had significantly more activation in brain regions involved in controlling the behavioral and autonomic aspects of the defensive response (e.g., periaqueductal grey, PAG) than rats exposed to cat odor in a group of four. However, as i.p.-wounded SENS mice did neither develop splenomegaly nor an increased basal and LPS-induced splenocyte in vitro viability, which is typical for bitten CSC mice developing splenic GC resistance, it remains to be investigated whether splenic GC resistance in i.p.-wounded SENS mice is also mediated by the invasion of GC insensitive myeloid cells as has been shown for bitten CSC mice (see introduction or^17,18^).

Noteworthy is finally that the lack of splenic GC resistance in SHAM-SENS mice of the present study is in line with earlier results showing that splenic GC resistance is detectable in mice physically exposed to the SDR paradigm but not in mice housed in sensory contact with conspecifics while being physically exposed to the SDR paradigm^47^, supporting again the critical role of bite wounding or experimental wounding in stress-induced GC resistance.

Taken together and considering that a number of above detailed future studies are still warranted, our findings support the hypothesis that housing male mice individually in continuous sensory contact with a CSC colony for 19 consecutive days and exposing these SENS mice to repeated standardized i.p.-wounding at Days 1, 8 and 15 of the chronic stress paradigm (i.e., matches the days when the dominant aggressor mouse is introduced/changed during CSC), represents an adequate tool to induce functional splenic in vitro GC resistance independent of the occurrence of uncontrollable bite wounds required in social stress paradigms to induce a comparable phenotype. As a complex experimental design like this, besides SENS mice with a highly comparable severity of splenic GC resistance, concurrently generates ordinary CSC mice characterized by varying bite wound severities, the latter can be used to investigate the mechanisms underlying bite-wound independent CSC-consequences, as for instance HPA axis changes or development of spontaneous colitis.

## Materials and methods

### Animals

Male C57BL/6N mice (experimental mice 17–19 g) and male CD-1 mice (dominant aggressor mice, 30–35 g) were purchased from Charles River (Sulzfeld, Germany). All mice were kept under standard specific pathogen free (SPF) laboratory conditions (12 h light/12 h dark cycle; lights on at 0600 h; 22 °C and 60% humidity) in standardized polycarbonate mouse cages (16 × 22 × 14 cm) and had free access to tap water and standard mouse diet. All experimental protocols were approved by the Committee on Animal Health and Care of the local government and performed in accordance with national and international guidelines on the ethical use of animals (ARRIVE guidelines and EU Directive 2010/63/EU for animal experiments). All efforts were made to minimize the number of animals used and their suffering. Given that the CSC paradigm is based on territorial aggression and establishment of a social hierarchy, only male mice were used in the present study.

### Experimental procedure

**Figure 3:**
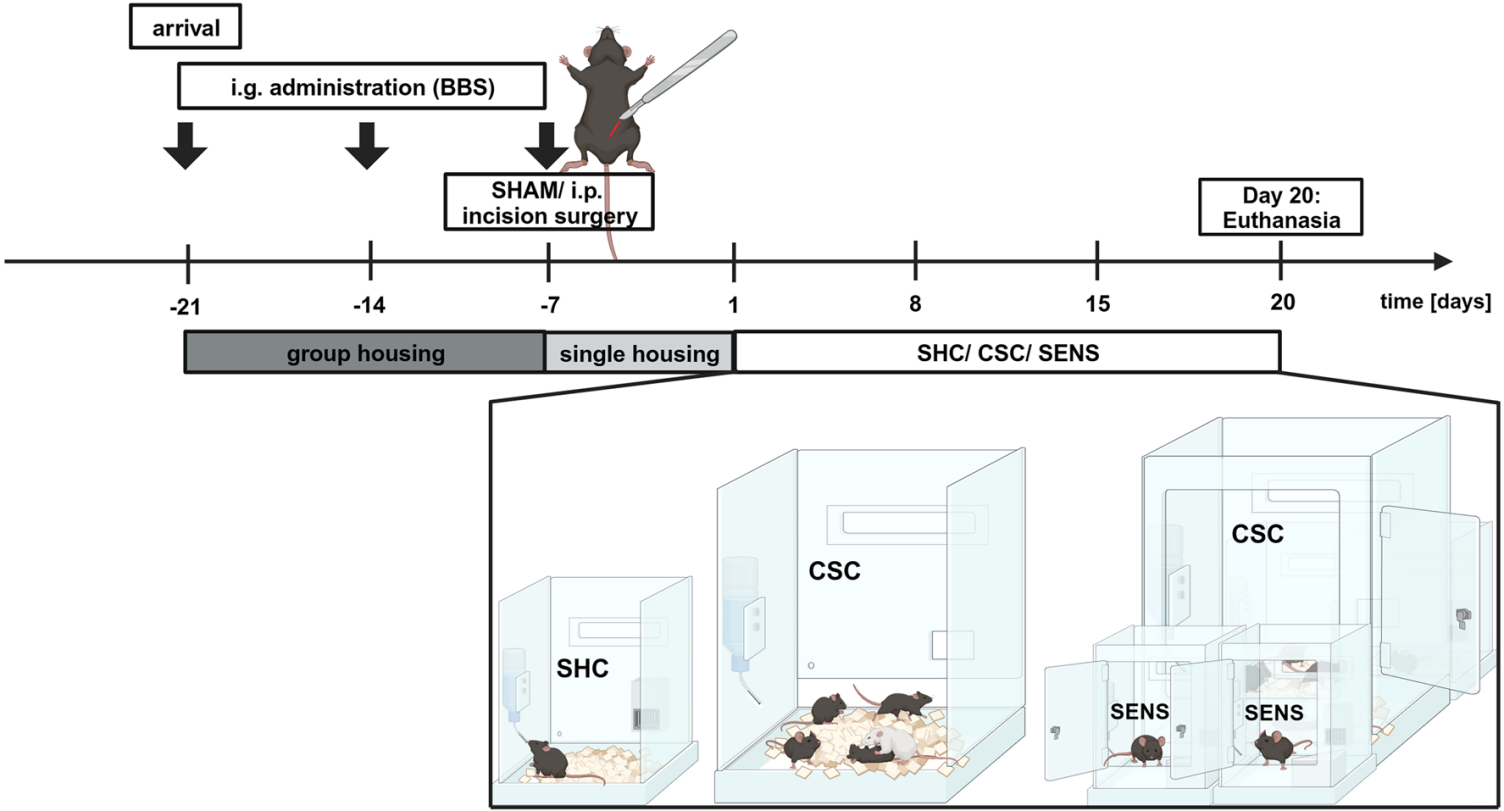
Experimental timeline. All experimental male C57BL/6N mice arrived at the animal facility on Day -21 and were kept in groups of four. On Days -21, -14 and -7, all mice were i.g. administered with BBS. Following the last administration on Day -7, mice were single-housed and either underwent a sterile i.p. incision or SHAM (i.e., anesthesia without wounding) surgery. Following one week of single-housing, experimental mice were either exposed to the CSC (19 Days) paradigm, kept single-housed (SHC) or were housed individually in SENS (i.e. visual, auditory and olfactory) contact with a CSC colony until the end of the experiment. Habituation of CSC mice to the dominant aggressor CD-1 was prevented by exposing CSC mice to a novel dominant CD-1 aggressor on Days 8 and 15. All experimental mice were euthanized on Day 20 for assessment of body, adrenal, thymus and spleen weights, bite wound severity (i.e., bite score) and splenic in vitro GC sensitivity in isolated and LPS-stimulated splenocytes*.* *i.g.* intragastrically, *BBS* borate-buffered saline, *i.p.* intraperitoneal, *CSC* chronic subordinate colony housing, *SHC* single-housed control, *SENS* sensory contact, *GC* glucocorticoid, *LPS* lipopolysaccharide.

Following arrival at Day -21, all experimental C57BL/6N mice were housed in groups of four and received repeated i.g. administrations of sterile borate-buffered saline (BBS) on Days -21, -14 and -7. Following the last i.g. administration on Day -7, all experimental mice were single-housed before either receiving a 1.0 cm-long i.p. incision wound under anesthesia (i.p.-group) or anesthesia only (SHAM-group). All mice were subcutaneously (s.c.) administered with analgesics during anesthesia and kept single-housed until the start of the CSC paradigm on Day 1. Bodyweight assessed on Day -7 was used to assign the animals either to the chronically-stressed CSC-group (SHAM-CSC: *N* = 12; i.p.-CSC: *N* = 12), the SENS-group (SHAM-SENS: *N* = 12; i.p.-SENS: *N* = 12), or the SHC-group (SHAM-SHC: *N* = 12; i.p.-SHC: *N* = 12). In the morning of Day 20, all experimental mice were again weighed and euthanized following brief CO_2_ inhalation for assessment of adrenal, thymus and spleen weights, severity of bite wounds as well as functional splenic in vitro GC resistance (Fig. 3).

### Intragastric (i.g.) administration of borate buffered saline (BBS)

Intragastric (i.g.) administrations of BBS were performed as described previously^29^. Briefly, 100 µL of BBS was administered via a 20-gauge gavage needle (Fine Science Tools Inc., Foster City, California, USA) on Days -21, -14 and -7.

### Sterile intraperitoneal (i.p.) wounding

Anesthesia and i.p. wounding was performed as previously described^22^ with slight modifications. Briefly, mice were anesthetized with 2.5% isoflurane/97% oxygen mixture under continuous flow of 0.5 L/min. Buprenorphine (Buprenovet® *sine* 0.3 mg/mL, Bayer, Richter Pharma AG, Wels, Austria) was administered s.c. (0.05 mg/kg body weight). The fur on the ventral left abdominal side was shaved and the skin underneath was disinfected. A 1.0 cm-long skin as well as peritoneal section (1 cm caudal of the diaphragm in cranial-caudal direction) was conducted on the ventral part of the left abdomen. Immediately afterwards, the abdominal incision was closed in layers (5–0 absorbable sutures; MonoPlus®, B. Braun Surgical, S.A Spain). Non-wounded SHAM mice underwent pre-surgery preparation (including anesthesia and analgesia) accordingly. I.p.-wounded and SHAM-treated mice were subsequently placed back into their individual home cages to recover.

### Chronic subordinate colony housing (CSC) paradigm

The chronic subordinate colony housing (CSC) paradigm was performed as previously described^16,30,32,48^. On Day 1, four experimental C57BL/6N mice (i.e., always two i.p.-wounded and two SHAM mice), were co-housed with a dominant male CD-1 mouse for 19 consecutive days. To avoid habituation, CSC mice were transferred to the home cage of a novel dominant aggressor CD-1 mouse on Days 8 and 15. Before the beginning of the CSC procedure, all potential dominant CD-1 mice were tested for their aggressiveness to exclude individuals severely injuring their conspecifics. SHC mice remained undisturbed in their home cages except for changing their bedding once a week. In a previous study, we convincingly demonstrated, that single housing is the adequate control group for the CSC paradigm, as group housing itself was shown to be stressful and to affect parameters assessed routinely in studies employing the CSC paradigm^49^.

### Sensory contact (SENS) paradigm

On Day 1, male C57BL/6N mice were placed in individual transparent cages separated from the adjacent CSC colony by perforated plexiglas partitions, allowing visual, olfactory and auditory to the CSC mice and the dominant aggressor CD-1 mouse^22^. All SENS mice were kept under this housing condition for 19 consecutive days.

### Assessment of the severity of bite wounds

Bite score assessment was performed as previously described^18,29^. Briefly, following decapitation, the skin (with fur attached) of the CSC mice was removed so that skin and body were still connected at the tail root and the legs. Afterwards pictures depicting both the skin (dermal) and the body (subdermal) of all CSC mice were taken using identical camera position and settings. All pictures were then digitally overlaid with a standardized grid consisting of 20 squares (4 × 5 squares; each square 180 × 240 pixels; overall 720 × 1200 pixels) covering the skin and 20 squares (4 × 5 squares; each square 180 × 240 pixels; overall 720 × 1200 pixels) covering the body. Afterwards the severity of the received bite wounds was scored according to a developed bite score, considering both size and intensity of wounds on the skin (dermal) and on the body (subdermal): body—affected area: 0, no bites; 1, < 1/3 of the area; 2, 1/3–2/3 of the area; 3 > 2/3 of the area; maximum subscore: 60. Body—intensity of injuries: 1, mild; 2, severe; maximum subscore: 40. Skin – affected area: 0, no bites; 1, single bite; 2, several bites; 3, spacious bites; 4, bites in the whole square; maximum subscore: 80. Skin—intensity of bite wounds: 1, mild; 2, moderate; 3, severe; 4, necrotic; maximum subscore: 80. Skin—degree of purulence: 0, no purulence; 1, mild purulence; 2, severe purulence; maximum subscore: 40. The total bite score of each mouse represents the sum of the skin (dermal) and the body (subdermal) and possible scores range from 0 to 300. Bite score assessment was performed by an investigator blinded to treatment.

### Determination of stress-sensitive organ weights

Following decapitation, adrenals, thymus and spleens were removed, pruned of fat and weighed. Spleens were subsequently stored in ice-cold Hanks’ balanced salt solution (HBSS; Sigma-Aldrich) until splenocyte isolation as described previously^18,19^. The thymus of one SHC-SHAM mouse had to be excluded due to tissue damage.

### Splenocytes isolation

The isolation of splenocytes was performed as previously described^17^ with minor modifications. In detail, spleens were mechanically disrupted using a nylon cell strainer (70 µm; Corning Inc., USA) and the plunger of a syringe to obtain a single cell suspension. Erythrocytes were removed by incubating the cell suspension for 2 min in lysis buffer (155 mM NH_4_CL, 10 mM KHCO_3_, 10 mM Ethylenediaminetetraacetic acid (EDTA)) followed by addition of Hanks’ Balanced Salt solution (HBSS; Gibco®, Thermo Fisher Scientific Inc.)/10% heat-inactivated fetal calf serum (FCS) to stop the lysis. Following one washing step with HBSS and filtration of the cell suspension through a 70 µm cell strainer, cells were resuspended in ice-cold Roswell Park Memorial Institute Medium (RPMI-1640, Gibco®, Thermo Fisher Scientific) containing 10% FCS, 50 U/mL penicillin and 50 µg/mL of streptomycin (RPMI sup.) and counted using an automated cell counter (ViCell XR, Beckman Coulter, Krefeld, Germany). For each sample the cell concentration was adjusted to 5 × 10^6^ cells/mL by adding the appropriate calculated volume of RPMI + 10% FCS + penicillin/streptomycin.

### Functional in vitro glucocorticoid (GC) sensitivity assay of isolated splenocytes

The functional in vitro GC sensitivity assay was performed as previously described^18,43^ with slight modifications. Briefly, isolated splenocytes (2.5 × 10^5^ cells/well) were stimulated with lipopolysaccharide (LPS; Escherichia coli O111:B4; final concentration: of 1.2 µg/mL, Sigma-Aldrich, Deisenhofen, Germany) or remained untreated to assess background activity (basal). For determination of GC sensitivity, unstimulated and LPS-stimulated cells were treated with different corticosterone (CORT; Sigma-Aldrich, Deisenhofen, Germany) concentrations (final concentrations: 0 and 5 µM) diluted in 95% ethanol. Cells were stimulated in flat bottom 96-well plates (final volume 100 µL/well) and incubated for 24 h (37 °C, 5% CO_2_). Afterwards, cell viability was measured by a commercially available colorimetric assay (CellTiter 96 Aqueous One Solution Cell Proliferation Assay, Promega, Madison, WI, USA) and the absorbance (optical density (OD)) of each well was measured at 450 nm using an enzyme-linked immunosorbent assay (ELISA) plate reader (Fluostar Optima, BMG Labtech GmbH, Offenburg, Germany). All values were blank corrected. The delta cell viability was calculated by subtracting the OD of unstimulated cells from the corresponding LPS-stimulated well and the resulting delta OD was set to 100% for the 0 µM CORT condition. A significantly lower delta cell viability of isolated splenocytes in the presence of CORT indicates a high GC sensitivity, while a high delta cell viability in the presence of CORT indicates a low GC sensitivity. A significantly increased delta cell viability of isolated splenocytes in the presence of CORT, independent of the dimension of the effect, in the current study is referred to as GC resistance. All samples were plated and measured in triplets and the mean of OD was used for statistical analysis. Due to technical issues, some values had to be excluded from statistical analysis, resulting in the following final group numbers: SHC-SHAM: *N* = 7, SHC-i.p.: *N* = 8; CSC-SHAM: *N* = 7, CSC-i.p.: *N *= 6; SENS-SHAM: *N* = 7, SENS-i.p.: *N* = 8.

### Statistical analysis

For statistical analysis and graphical illustrations GraphPad Prism (Version 9.4.0, GraphPad Software, LCC) was used. Kolmogorov–Smirnov test with Lilliefors’ correction was employed to test for normal distribution. Normally distributed data sets were tested for outliers employing Grubbs test^50^. Normally distributed data sets were analyzed by parametric statistics, i.e., two-way ANOVA (two factors, two or more independent samples). All statistical tests comparing more than two samples were followed by post hoc analysis using Bonferroni pairwise comparison. Non-normally distributed data sets were analyzed by non-parametric statistics, i.e. Mann–Whitney *U* test (MWU; one factor, two independent samples) and Wilcoxon matched-pairs signed rank test (one factor, two dependent samples). Data are presented as mean + SEM including individual values. The level of significance was set at *P* ≤ 0.05.

## Data Availability

The datasets generated and analysed in the current study are available from the corresponding author on reasonable request.
